# Electro-Metabolic Coupling of Cumulus–Oocyte Complex

**DOI:** 10.3390/ijms25105349

**Published:** 2024-05-14

**Authors:** Diletta Del Bianco, Rosaria Gentile, Luana Sallicandro, Andrea Biagini, Paola Tiziana Quellari, Elko Gliozheni, Paola Sabbatini, Francesco Ragonese, Antonio Malvasi, Antonio D’Amato, Giorgio Maria Baldini, Giuseppe Trojano, Andrea Tinelli, Bernard Fioretti

**Affiliations:** 1 Department of Chemistry, Biology and Biotechnologies, University of Perugia, Via dell’Elce di Sotto 8, 06132 Perugia, Italy; diletta.delbianco@dottorandi.unipg.it (D.D.B.); rosaria.gentile@dottorandi.unipg.it (R.G.); luana.sallicandro@dottorandi.unipg.it (L.S.); andrea.biagini@dottorandi.unipg.it (A.B.); paolatiziana.quellari@dottorandi.unipg.it (P.T.Q.); elkogliozheni@gmail.com (E.G.); paola.sabbatini@unipg.it (P.S.); francesco.ragonese@unipg.it (F.R.); 2 Laboratorio Interdipartimentale di Fisiopatologia della Riproduzione, Università degli Studi di Perugia, Edificio C, Piano 3 P.zza Lucio Severi, 1, Sant’Andrea delle Fratte, 06132 Perugia, Italy; 3 Department of Medicine and Surgery, Perugia Medical School, University of Perugia, Piazza Lucio Severi 1, 06132 Perugia, Italy; 4 ASST Grande Ospedale Metropolitano Niguarda, 20162 Milano, Italy; 5 Department of Obstetrics and Gynecology, Faculty of Medicine, University of Tirana, AL1005 Tirana, Albania; 6 Department of Biomedical Sciences and Human Oncology, University of Bari, 70121 Bari, Italy; antoniomalvasi@gmail.com; 7 1st Unit of Obstetrics and Gynecology, University of Bari, 70121 Bari, Italy; antoniodamato19@libero.it; 8 MOMO Ferti LIFE IVF Center, 76011 Bisceglie, Italy; gbaldini97@gmail.com; 9 Department of Maternal and Child Health, “Madonna delle Grazie” Hospital ASM, 75100 Matera, Italy; giuseppe.trojano@asmbasilicata.it; 10 Department of Obstetrics and Gynecology and CERICSAL (CEntro di RIcerca Clinico SALentino), Veris delli Ponti Hospital, Via Giuseppina delli Ponti, 73020 Scorrano, Lecce, Italy

**Keywords:** glucose metabolism, cumulus cells, oocyte, gap junctions, resveratrol

## Abstract

Oocyte–cumulus cell interaction is essential for oocyte maturation and competence. The bidirectional crosstalk network mediated by gap junctions is fundamental for the metabolic cooperation between these cells. As cumulus cells exhibit a more glycolytic phenotype, they can provide metabolic substrates that the oocyte can use to produce ATP via oxidative phosphorylation. The impairment of mitochondrial activity plays a crucial role in ovarian aging and, thus, in fertility, determining the success or failure of assisted reproductive techniques. This review aims to deepen the knowledge about the electro-metabolic coupling of the cumulus–oocyte complex and to hypothesize a putative role of potassium channel modulators in order to improve fertility, promote intracellular Ca^2+^ influx, and increase the mitochondrial biogenesis and resulting ATP levels in cumulus cells.

## 1. Introduction

**Figure 1:**
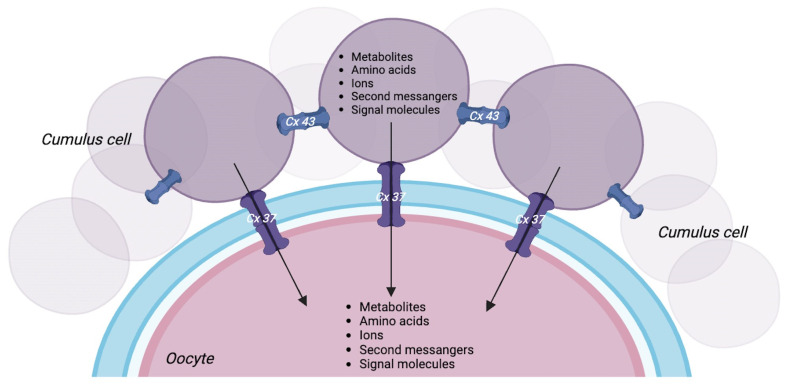
Bidirectional cumulus–oocyte relationship. A model showing intercellular communication in the cumulus–oocyte complex (COC). The cellular crosstalk between the oocyte and the surrounding somatic cells is mediated by communication through gap junctions, allowing the passage of low-molecular-weight molecules. Created with BioRender.com (accessed on 25 January 2024).

The oocyte is surrounded by cumulus cells (CCs) in an integrated system, which is essential for oocyte competence. During various stages of folliculogenesis, oocytes grow together with the surrounding granulosa cells (GCs) and progressively acquire increasing competence [1], which depends on several factors such as stimulation from gonadotropins and communication between the oocyte and the CCs surrounding it [2]. Communication between the oocyte and the CCs is made possible by cytoplasmic projections that pass from the CCs through the zona pellucida and form gap junctions, giving origin to the cumulus–oocyte complex (COC) [3]. Gap junctions are intercellular membrane channels composed of connexin (Cx), a family of integral membrane proteins, including connexin 37 (Cx37), connexin 43 (Cx43), and connexin 45 (Cx45). Cx37 maintains a bidirectional relationship between oocyte and cumulus cells, while Cx43 is required to maintain a connection between the granulosa cells themselves and between GCs and CCs [4,5]. Gap junctions allow the direct passage of some low-molecular-weight molecules such as ions, metabolites, amino acids, and intracellular signaling molecules from the CCs to the oocyte [6,7] (Figure 1). Cx43 is widely expressed at the level of GCs during all follicular stages, and its expression is essential for GC proliferation [8,9]. It is known that Cx43 is present during all stages of folliculogenesis, but its expression increases as the follicle develops and matures [10]. Cx45 is expressed together with Cx43 but differs from it because its expression is independent of the follicle growth [10,11]. From studies carried out on ovary knockout for Cx43 and Cx37, failure of folliculogenesis was observed, confirming the importance of the connexins for oocyte and GG maturation [9,12]. Furthermore, the loss of function of the gene encoding for Cx37 leads to a lack of communication between the oocyte and the surrounding CCs, thus determining the interruption of follicle development in the antral phase, non-competent oocytes, and ovulatory dysfunction, causing infertility in mice [12]. This shows that communication between the oocyte and CCs is essential to ensure proper oocyte development, ovulation, and competence [13].

## 2. Gap Junction in Cell Signaling

**Figure 2:**
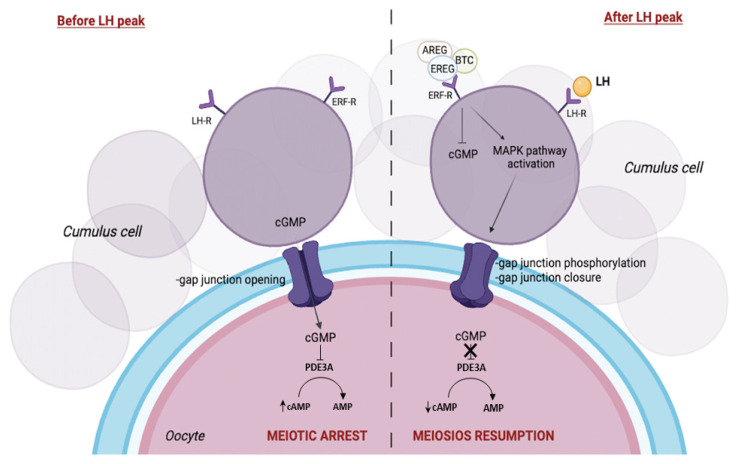
Regulation of oocyte maturation. Schematic diagram of oocyte meiotic arrest in the phase preceding the gonadotropic peak (**left**) and gonadotropin-induced oocyte meiotic resumption (**right**). Meiotic regulation is modulated by the levels of cyclic guanosine monophosphate (cGMP) and Adenosine 3′,5′-cyclic monophosphate (cAMP) that are transferred from cumulus cells (CCs) to the oocyte. Created with BioRender.com (accessed on 25 January 2024).

Oocyte development, maturation, and ovulation are highly interconnected events that are regulated by endocrine signals and surrounding somatic cells [14]. Ovulation is triggered after release by the pituitary gland of the luteinizing hormone (LH). LH, by binding to G-protein-coupled receptor (LHR) expressed at the level of GCs [14,15], promotes oocyte maturation and subsequent follicle rupture [16,17,18]. It is well known that the stimulus of LH reaches the oocyte via secondary molecules belonging to the epidermal growth factor (EGF) family. The EGF family includes 11 proteins, including EGF, anfiregulin (AREG), epiregulin (EREG), and betacellulin (BTC) [19]. Specifically, in human ovarian follicular cells, the expression of EGF-like factors appears to be mediated by activation of the second messenger 3′,5′-cyclic adenosine monophosphate (cAMP) after stimulation with LH [20,21]. Therefore, the production of EGF-like peptides, such as AREG, EREG, and BTC, represents an important paracrine component of ovulatory LH signaling that acts by coordinating various processes such as oocyte maturation, cumulus expansion, and ovulation [22]. It has been observed that epidermal growth factor receptors (EGFR) are expressed at the level of GCs and CCs [23,24,25]. Interestingly, these processes are lost in AREG or EREG knockout mice [26,27,28]. EGF-like peptides (AREG, EREG, and BTC) are localized at the outer plasma membrane of GCs as inactive transmembrane protein [19]. EGF-like activation occurs by proteolytic cleavage promoted by ectoprotease, a disintegrin, and metalloprotease 17 (ADAM17), also called TACE, in order to release the soluble extracellular EGF domain [29]. It is known that the follicle-stimulating hormone (FSH) and LH promote the upregulation of TACE in the COC [30]. The active peptides, once released into the antral fluid, bind to EGFR located on the GCs and CCs in an autocrine and paracrine manner. Therefore, the ovulatory signal of the LH/EGF peptide, via EGFR present on CCs, is transferred to the oocyte [31]. It is well known that EGF induces meiosis resumption in oocytes [32,33]. The signal transduction in follicular somatic cells regulates oocyte meiosis in response to LH peak. In fact, in the phase preceding the gonadotropic surge, meiotic progression is blocked by the action of cyclic guanine 3′-5′ monophosphate (cGMP), which, passing into the oocyte through gap junctions, inhibits the hydrolysis of cAMP by phosphodiesterase (PDE3A) [34]. Thus, high levels of cAMP necessary for the maintenance of meiotic arrest are present within the oocyte [35]. In contrast, in the phase preceding ovulation, stimulation by LH causes in somatic cells a reduction in cGMP level and its subsequent reduction in the oocyte via gap junction closures [35]. The absence of the inhibitory signal leads the oocyte to a reduction in cAMP levels and resumption of meiosis [35]. Therefore, EGF-like factors, via the EGFR, may act as intrafollicular mediators for the resumption of oocyte meiosis via activation of the mitogen-activated protein kinase (MAPK) pathway [20]. The molecular mechanism involves the release of LH that induces MAPK to activate and promote Cx43 phosphorylation. This connexin is one of the protein components of the gap junctions between the granulosa cells themselves and between them and the CCs, resulting in the interruption of the gap junctions [36,37,38]. The lack of communication between GCs and CCs in turn fosters a reduction in the interaction between the CCs and the oocyte, resulting in decreased cAMP and resumption of meiosis in the oocyte [39] (Figure 2). EGF-like factors also downregulate cGMP production, which has recently been shown to be necessary for the resumption of oocyte meiosis [20].

## 3. Gap Junction and Metabolism

Metabolic cooperation is known to exist between the oocyte and the surrounding CCs. Although CCs are removed in assisted reproduction techniques, they are useful for assessing oocyte quality [40], as several studies have shown a close correlation between the mitochondrial status of CCs and oocyte quality [41,42,43]. CCs influence intraoocyte ATP levels, as oocytes enclosed in CCs matured in vitro express higher ATP concentrations than oocytes matured without the CC lining (denuded oocytes) [42,44]. Interestingly, gap junctions, in particular Cx43, provide a means for the transfer of intercellular organelles, including mitochondria. During the mitochondrial transfer process, one of the two cells phagocytizes the gap junctions by invading the membrane and cytoplasm of the neighboring cell with the formation of a double-membrane vesicle called a connexosome or annular gap junction. The process of internalization in ovarian follicles is not fully understood but could also be a useful means of sending mitochondrial metabolic products to neighboring recipient cells [45].

### 3.1. Glycolysis

**Figure 3:**
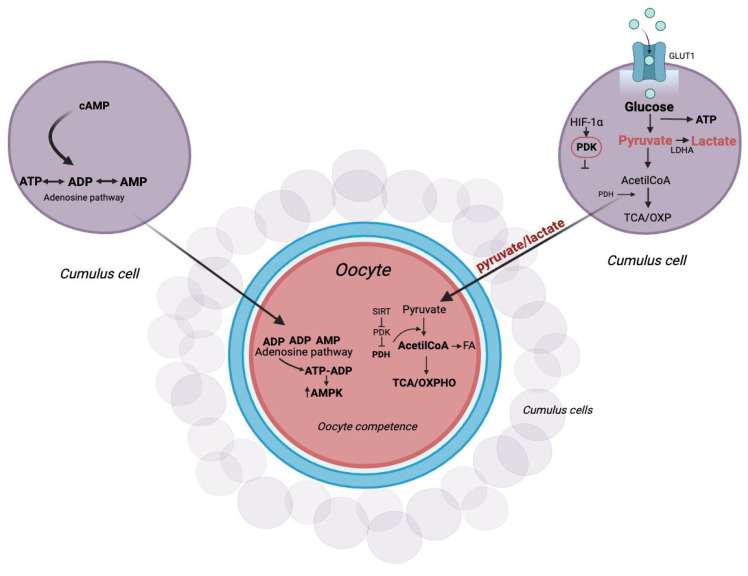
Cumulus–oocyte metabolic coupling. Cumulus and oocyte cell metabolic reprogramming is dependent on the pyruvate dehydrogenase kinase (PDK) and pyruvate dehydrogenase (PDH) enzymes. The schematic illustration shows the molecular mechanisms of glucose metabolism within the COC. Metabolic cooperation between the two cell types is also made possible by the transfer of Adenosine triphosphate (ATP) generated by the heap via glycolysis and the adenosine salvage pathway. Created with BioRender.com (accessed on 25 January 2024).

Among metabolites, glucose plays an essential role in both cumulus cell expansion and oocyte maturation [46]. The energy metabolism of mammalian folliculogenesis and embryogenesis is based on glycolysis, the tricarboxylic acid cycle or Krebs cycle, and, lastly, oxidative phosphorylation (OXPHOS) [47]. Glycolysis is a process that promotes the metabolization of glucose into cytoplasmic pyruvate, which, in turn, can be converted into lactate under anaerobic conditions. Via glycolysis, 2 ATP molecules are generated for each glucose molecule; in contrast, OXPHOS has a high energy yield as it generates 36 ATP molecules [48]. Oocytes have a low capacity to recruit glucose [48] and induce glycolysis [49,50] because they have a low expression of the enzyme phosphofructokinase (PFK), one of the rate-limiting enzymes for glycolysis [51]. However, CCs and oocytes provide energy to the process of oogenesis via different metabolic pathways [3]. Therefore, CCs metabolize glucose via glycolysis, generating ATP and the products of glycolysis, such as pyruvate and lactate. The glycolytic products are then supplied to the oocyte, which metabolizes them in order to generate ATP via mitochondrial OXPHOS to promote oocyte competence [52]. However, pyruvate can also be metabolized by the CCs themselves via the tricarboxylic acid cycle followed by OXPHOS to produce much higher amounts of ATP than glycolysis [53]. In order to facilitate the supply of glycolytic products from CCs [49], oocytes secrete paracrine factors such as growth differentiation factor 9 (GDF-9) and bone morphogenetic protein 15 (BMP-15), which induce the expression of primary glycolytic genes [54]. These findings could suggest a metabolic co-dependence between oocytes and CCs [52]. An essential role in the metabolism of CCs and oocytes is played by metabolic enzymes such as pyruvate dehydrogenase kinase (PDK), pyruvate dehydrogenase (PDH), and lactate dehydrogenase (LDH). In mouse cumulus cells, a glycolytic phenotype was observed [55], as the expression of the enzyme PDK mitochondrial kinase blocks PDH activity by preventing the conversion of pyruvate to acetyl-CoA [55]. This over-expression of the PDK enzyme in CCs leads to the formation of pyruvate and lactate, which, once transferred to the oocyte, acts positively on mitochondrial activity and consequently on ATP production. In this way, the expression of mitochondrial PDH in the oocyte decarboxylates pyruvate into acetyl-CoA, thus shifting the metabolism toward OXPHOS, which is essential for the proper development of the oocyte itself. Furthermore, acetyl-CoA can be produced not only by the PDK-PDH axis but also from fatty acids catabolism that implements ATP production during mitochondrial OXPHOS [56]. Therefore, the beta-oxidation process acts by improving the rate of oocyte maturation [56]. Glycolytic metabolism, promoted by the action of PDK, can inhibit reactive oxygen species (ROS) formation and thus spare CCs from apoptosis [57]. At the same time, prolonged activation of PDK via suppression of OXPHOS inhibits ATP production (Figure 3). This process causes an increase in apoptosis, resulting in the cessation of CC proliferation [58]. The metabolic shift toward glycolysis in CCs is also shared by cancer cells via a process known as the “Warburg effect” [52,59], which is thought to be a metabolic adaptation to hypoxia [49,60,61]. This is confirmed by gene expression analysis of CCs, in which differential expression of the gene encoding for the hypoxia-inducible factor (HIF-1α) is observed [62]. This is implicated in the over-expression of glycolytic enzymes such as glucose transporter (GLUT1), PDK, and LDH, thus inducing a metabolic shift from OXPHOS to glycolysis [63]. Therefore, PDK enzyme activity appears to be dependent on the oxygen levels present in the follicle or in the COC [64].

Sirtuins are a family of proteins that act as metabolic sensors by modifying histones and proteins via post-translational modifications in response to changes in the metabolic state. Sirtuins perform various functions, including regulation of aging and mitochondrial function (Figure 3), DNA repair and recombination, microtubule organization, and play a role in gene and epigenetic silencing [65,66,67]. They are deacetylating proteins dependent on nicotinamide adenine dinucleotide (NAD)/NADH levels, so they are responsive to the cellular metabolic state [68]. The activity of SIRT5, a mitochondrial protein present in GCs and CCs, is reduced in older women with diminished ovarian reserve [69]. Another mitochondrial protein, SIRT3, can detect a change in metabolic state and modify mitochondrial function. SIRT3 is mainly expressed in mitochondria-rich tissues [70,71,72] and, depending on the different tissue types, can be localized within the mitochondria or in both the nucleus and mitochondria [70,73]. SIRT3 promotes enzyme deacetylation of complexes I, II, and IV of the electron transport chain, thereby increasing OXPHOS [72,74,75,76,77]. Since the oocyte requires follicular metabolism for the acquisition of competence, modifications of SIRT3 and its targets in the GCs and CCs may cause an alteration of the follicular environment and consequently may affect oocyte health [78]. Also, SIRT6 is associated with reduced expression of HIF-1α, resulting in the downregulation of glycolytic genes and upregulation of mitochondrial respiration [79]. Thus, sirtuins maintain metabolic homeostasis via the regulation of epigenetic modifications [80]. Therefore, metabolic changes in CCs and oocytes may be regulated by genetic and epigenetic changes that occur at the tricarboxylic acid cycle enzyme level.

### 3.2. Pentose Phosphate and Other Pathways

It is known that glucose can be metabolized via pathways other than glycolysis, for example, via the pentose phosphate pathway (PPP) and the hexosamine biosynthesis pathway (HBP). The PPP represents the additional route by which a small amount of glucose can be metabolized by CCs [53]. PPP consists of two phases: one oxidative and one non-oxidative. In the oxidative phase, the oxidation of glucose 6-phosphate to ribose 5-phosphate sugar leads to the production of nicotinamide adenine dinucleotide phosphate (NADPH), which is essential because it becomes part of anabolic pathways, such as nucleotide synthesis, and participates in the reduction in oxidized glutathione by promoting the formation of reduced glutathione (GSH). The GSH produced represents an important antioxidant in the oocyte that protects against damage by ROS formed as by-products of mitochondrial respiration. ROS formation, during metabolism, is favorable for oocyte maturation, but conversely, an excessive production can create damage to cellular molecules and affect oocyte quality [81]. Confirming this, oocytes from older women have been shown to exhibit reduced expression of antioxidant enzymes [82], causing altered oocyte maturation [81]. Therefore, decreased ROS production and increased GSH as an antioxidant defense are critical for oocyte maturation because oocyte aging and apoptosis are inhibited [53,83,84]. Indeed, in women undergoing in vitro fertilization, it has been demonstrated that an elevated level of GSH within the follicles is associated with an enhanced fertilization rate [85]. PPP produces substrates useful for the synthesis of nicotinamide adenine dinucleotide (NAD), a cofactor and enzyme substrate required for a wide variety of vital cellular processes. However, ribose-5 phosphate, generated by this pathway, is a precursor for the synthesis of NAD^+^. This cofactor is important because it can positively modulate the catalytic activity of sirtuins, which are implicated in many metabolic and cellular aging processes [86]. Recently, by analyzing aged mouse oocytes, the treatment with metabolic NAD^+^ precursors was shown to restore oocyte quality and fertility [87]. Therefore, the PPP positively influences oocyte quality via the presence of antioxidants and DNA repair mechanisms by increasing NAD^+^/sirtuin. The HBP is another route by which glucose can be metabolized. The end product of HBP is UDP-N-acetylglucosamine, which is used to produce hyaluronic acid glycosaminoglycan, which is secreted by CCs to form a viscoelastic matrix necessary for its own expansion in order to obtain oocyte maturation [88,89,90]. Proper expansion of the CC is essential for ovulation [91] since it facilitates the ejection of the oocyte, transport to the fertilization site, and subsequent penetration by sperm [92]. Therefore, CC expansion and CC matrix constituents are related to the competence of oocyte development [93,94]. Another example of metabolic support concerns the uptake of certain amino acids, such as L-alanine, which are considered “coupling-dependent” amino acids because they are first uptaken by the CCs and then transferred to the oocyte via gap junctions. This has been observed because when oocytes are cultured with radio-labeled L-alanine, the amount of radioactivity is greater in the oocytes enclosed by the CCs than in the denuded ones [95]. However, there are also “non-coupling-dependent” amino acids, such as L-leucine, that are incorporated regardless of the presence or absence of CCs around the oocytes [95]. In fact, by studying murine CCs, it was seen that gene expression is very similar to that of GCs, even if the transcriptome of cumulus cells has a unique profile consistent with their function. For example, the expression of the Slc38a3 gene, which encodes for a sodium-coupled neutral amino acid transporter, was found to be restricted to CCs and appears to require close binding to the oocyte to enhance cooperation in amino acid transport between the two cell types.

## 4. Gap Junction and ATP

### 4.1. The Role of Mitochondria

Mitochondria are directly involved in the energy metabolism of the cell [96] because via OXPHOS, they provide large amounts of ATP, which are useful to perform numerous cellular functions. Mitochondrial OXPHOS relies on the activity of five multienzyme complexes, the first four of which (complexes I–IV) constitute the electron transport chain, while the fifth complex (complex V) is represented by ATP synthase, which produces energy in the form of ATP. Mitochondrial energy function is also regulated by mitochondrial permeability transition pores (mtPTPs), acting as sensors. Specifically, when there is a decrease in energy level, the pores open, triggering cellular apoptosis. Therefore, mitochondrial dysfunction that inhibits OXPHOS causes a reduction in ATP, generates ROS as by-products, and leads to apoptosis [96]. The mitochondrial genome is represented by mitochondrial DNA (mtDNA), a circular double-stranded molecule consisting of 16.569 bp [97]. Mature oocytes contain several hundred thousand copies of mtDNA, depending on the species to which they belong [98] because, during the late stage of the oogenesis process, female germ cells acquire a large amount of mitochondrial mass [99,100,101,102] in addition to other components. The number of copies of mtDNA in CCs affects the competence of the oocyte and consequently may be informative for the purpose of in vitro fertilization (IVF) as it “selects” quality embryos [103,104]. However, mtDNA copy number may reflect mitochondrial biogenesis and is a surrogate marker of its function [105]. Defective mitochondrial biogenesis in CCs can modify both the oocyte and the CCs [106]. In fact, mitochondrial function can be studied via the concentration of intracellular ATP that is produced, and to confirm this, at-risk fertilization and embryonic development have been observed when ATP is insufficient [107,108]. Examination of CCs, therefore, can be a valuable aid in providing information on the metabolic processes underlying ovarian dysfunction caused by aging [41,109]. The concentration of ATP in CCs was assessed in two groups consisting of young and old women, respectively, and from the results, it was shown that ATP levels in the young women’s group were about 4.3 times higher than that in the old women’s group [110]. This observation may confirm how in aged women, energy production in CCs is reduced, thus affecting their fertility. Therefore, CCs affect intra-oocyte ATP levels; in vitro studies showed that oocytes enclosed in matured CCs express higher ATP concentrations than oocytes matured without the CC lining (denuded oocytes) [42,44]. Furthermore, the decrease in ATP in oocytes may also be caused by the closure of gap junctions between the oocyte and CCs [42], suggesting that CCs provide the oocyte with energy support in the form of energy substrates and ATP. However, several observations suggest that the CCs can also generate ATP via the adenosine rescue pathway and then supply it directly to the oocyte through the gap junctions [111] or simply by supplying adenosine monophosphate (AMP) obtained from the degradation of cAMP to the oocyte which will then use it to form ATP [44]. This pathway is a two-step enzymatic process in which AMP can be phosphorylated to adenosine diphosphate (ADP) by adenylate cyclase, and ADP is phosphorylated to ATP by creatine kinase. Therefore, when the oocyte, during its maturation, still has immature, hooded mitochondria containing fewer ridges, it can use this adenosine rescue pathway to produce ATP as an alternative to OXPHOS [112]. AMP, ADP, and ATP are also modulators of AMP-activated protein kinase (AMPK), which acts as a nutrient and sensor in order to maintain energy homeostasis [113]. Human primordial oocytes originate during fetal development and remain in a dormant state for up to 50 years. During this long period of quiescence, oocytes maintain the ability to generate a new organism after fertilization [114,115,116] because they inactivate mitochondrial complex I while maintaining the remaining complexes of the OXPHOS system functioning [117]. In this way, by turning off complex I, oocytes can continue the biosynthesis reactions of essential biomolecules [118], keeping their mitochondrial activity low to avoid the production of ROS [117]. It is recognized that ROS are formed as by-products of mitochondrial OXPHOS and are associated with lower fertilization rates and embryo survival rates [114,115,116]. ROS, at low concentrations, are functional since they act as signaling molecules [119]; on the contrary, their high concentration favors DNA mutagenesis, leading to cellular apoptotic mechanisms. Therefore, the concentration of ROS is correlated to reduced oocyte competence [114,115,116]. Via functional imaging techniques, it has been demonstrated that the mitochondrial membrane potential in human oocytes is lower than that of the surrounding GCs [117]. In fact, the analysis revealed that the activity of the mitochondrial electron transport chain in early oocytes is low [117]. However, it can be concluded that complex I is absent in early oocytes but present and perfectly functional in maturing and late-stage oocytes [117].

### 4.2. The Relationship between Voltage Resting Membrane Potential and Mitochondrial Biogenesis

**Figure 4:**
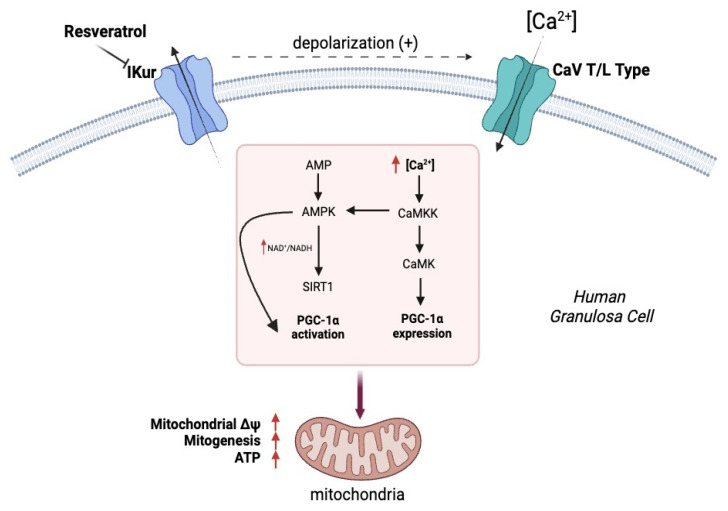
Mitochondrial biogenesis. The schematic diagram shows how resveratrol decreases the functional expression of voltage-dependent potassium currents by causing a depolarization of the cell membrane in human ovarian granulosa cells (hGCs). This event promotes an increase in intracellular Ca^2+^ that leads to an improvement in mitochondrial function with an increase in mitochondrial biogenesis. Created with BioRender.com (accessed on 25 January 2024).

Intracellular calcium (Ca^2+^) plays a crucial role because it has been shown that voltage dependent Ca^2+^ channels are required for the purposes of gene expression, neurotransmission, and other physiological responses [120]. Also, it has been demonstrated how abnormal expression of these channels can be closely related to many diseases [121]. Recently, certain substances, such as resveratrol, have been shown to induce an increase in intracellular Ca^2+^ at the level of mural granulosa cells (MGC) [122]. The influx of intracellular Ca^2+^ is due to a decrease in membrane potassium conductance promoted by resveratrol, resulting in a depolarization of the membrane. Subsequently, this promotes the opening of L-type and T-type transmembrane-dependent Ca^2+^-voltage channels present in GCs [122,123,124]. Voltage-dependent potassium (Kv) channels are critical to setting resting membrane potential in a complexity of cells [122,125]. Interestingly increased intracellular Ca^2+^ may promote mitochondrial biogenesis with an ultimate improvement in the energy metabolism of the cell. However, treatment of GCs at 48 h with ionomycin, an ionophore produced by the bacterium Streptomyces conglobatus, increases mitochondrial biogenesis [126]. The relationship between mitochondrial biogenesis and increased intracellular Ca^2+^, dependent on activation of the β Ca/calmodulin-dependent protein kinase/AMPK/SIRT1 pathway, promotes the expression of peroxisome proliferator-activated receptor gamma coactivator 1α (PGC-1α), resulting in a stimulation of mitochondrial biogenesis [127] (Figure 4). All these results suggest the central role of ionic channels in the control of the granulosa cell functionality, in particular of the potassium channels, based on their critical role in the resting membrane potential setting. In this context, the efficacy of resveratrol in promoting mitochondrial biogenesis in primary and immortalized GCs after 48 h treatment was also observed [122]. Resveratrol (3,5,4′-trihydroxystilbene) is a natural polyphenol found in peanuts, red grape skins, and red wine [128], synthesized by plants as phytoalexin in response to attacks by pathogens such as bacteria or fungi [129]. This polyphenol is known to possess antioxidant, anti-inflammatory, and antithrombotic effects [130,131]. In a study conducted on women with polycystic ovary syndrome (PCOS), an endocrine–metabolic disease affecting women of childbearing age [132], characterized by ovulatory dysfunction and other clinical symptoms, resveratrol was shown to have beneficial effects on PCOS symptomatology [133]. Environmental factors such as tributyltin (TBS), a chemical substance generally applied as a biocide that acts as an endocrine disruptor, are known to intervene in the etiopathogenetic process of PCOS [133], going on to create damage to transjunctional projections (TZPs), structures involved in COC communication, reported to be important for oocyte quality and competence [133]. In a mouse knock-out model of myosin-X (MYO10), a structural component of TZPs, it was observed that the absence of MYO10 causes a reduction in TZP density, resulting in altered gene expression in oocytes lacking TZP [134]. This study showed that the reduction in TZPs affects oocyte maturation and subsequent early embryo development with reduced fertility in mice [134]. Thus, TZPs have the function of maintaining intact the structure of the germinal-somatic complex necessary for the regulation of gene expression in the oocyte and thus for its development [134]. Several studies have shown that the TZPs of patients with PCOS are much weaker than those of healthy women [135,136]. In this context, resveratrol would resolve the damage to TZPs by ameliorating TBS-induced PCOS via the transport of calcium ions into the cytosol and the subsequent activation of Ca β Ca/calmodulin-dependent protein kinase II β (CaMKIIβ) [133]. This is critical in maintaining the stable, rigid actin filament system that makes up TBTs [137]. Previous studies have shown that under conditions of increased Ca^2+^, Ca^2+^ activates calmodulin, which, via phosphorylation of CaMKIIβ, allows the disjunction of CaMKIIβ from actins [138], making them available to polymerize and form TZPs, which are essential for proper oocyte maturation. To prove this, patients treated with TBT show lower Ca^2+^ levels accompanied by lower CaMKIIβ phosphorylation levels with failure to polymerize actin filaments [133]. However, even in a study on the human ovarian granulosa-like tumor cell line (KGN) under hypoxic conditions, it was suggested that resveratrol improves mitochondrial quantity by activating the SIRT1/PGC-1α signaling pathway. SIRT1 [139] is controlled by NAD/NADH levels, which acts by deacetylating peroxisome proliferator-activated receptor gamma coactivator 1α (PGC-1α) [140,141]. PGC-1α is recognized to be a regulator of energy metabolism [142]. Therefore, SIRT1 and PGC-1α are involved in mitochondrial biogenesis, and reduced SIRT1 activity inhibits PGC-1α [143,144,145]. Although the role of resveratrol in hypoxic stress in the ovary remains unclear, it is recognized that, under hypoxic conditions, the expression of SIRT1 and PGC-1α mRNA is upregulated, while the expression of HIF-1α, which regulates the expression of angiogenic genes such as vascular endothelial growth factor (VEGF), is stabilized [144]. Resveratrol also shows promise in endometriosis, a condition that impairs embryo implantation, due to its anti-inflammatory and anti-angiogenic effects [146]. In fact, this polyphenol acts by inhibiting the expression of certain inflammatory biomarkers such as tumor necrosis factor alfa (TNFα) and cyclooxygenase-2 (COX-2) and induces antioxidant enzymes in order to counteract the chronic inflammation that characterizes this pathology [146]. In addition, its anti-angiogenic effect is expressed by blocking VEGF, which influences endothelial cell proliferation, migration, and permeability [146]. Therefore, via the induction in SIRT1 and PGC-1α expression, resveratrol acts by significantly increasing mtDNA copy number. However, the effects of resveratrol on potassium current, increased intracellular Ca^2+^, and mitochondrial biogenesis in MGC could explain the positive effects of this polyphenol on the overall physiology of the female reproductive system, suggesting potential therapy in clinical settings.

### 4.3. Gap Junction and Electrical Coupling of COC

**Figure 5:**
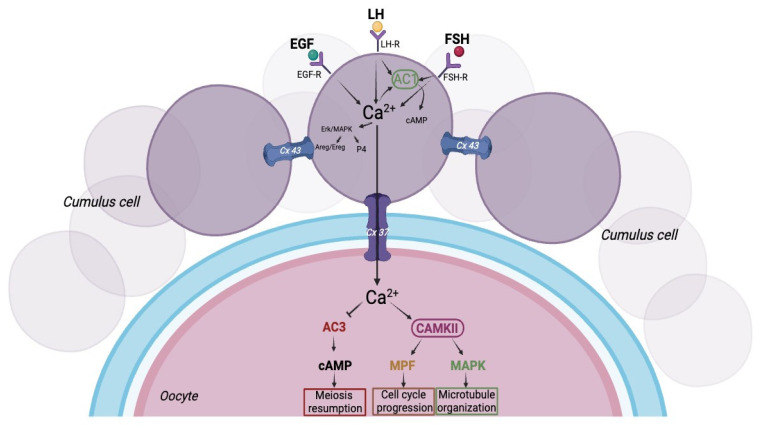
Role of intracellular calcium in oocyte maturation. Follicle-stimulating hormone (FSH), luteinizing hormone (LH), and epidermal growth factor-like (EGF-like) paracrine factors bind to their receptors on the cumulus cell and induce intracellular Ca mobilization. The increase of intracellular Ca^2+^ in the CCs can be transmitted to the oocyte through gap junctions. In the oocyte, Ca can inhibit adenylyl cyclase isoform 3 (AC3), resulting in a reduction in cAMP in the oocyte. Alternatively, Ca^2+^ can activate Ca/calmodulin-dependent protein kinase II (CAMKII), which in turn activates Maturation Promoting Factor (MPF) and mitogen-activated protein kinase (MAPK), promoting cell cycle progression and spindle formation important for oocyte maturation. Created with BioRender.com.

It appears that the oocytes, in addition to being metabolically and hormonally coupled with the surrounding CCs, are also electrically coupled. These couplings turn out to be necessary for oocyte maturation; in fact, it has been reported that the membrane potential of the oocyte is regulated by the surrounding CCs [147]. Oocytes and CCs are found to have different membrane potentials when they are not connected to each other; in contrast, when they are in the form of COC, electrical coupling occurs [148]. In fact, the results obtained from this study show that oocytes enclosed in CCs have a resting membrane potential of about −40 mV, while oocytes deprived of CCs (denuded oocytes) have a resting membrane potential of about −30 mV [147]. This suggests that the membrane potential of the two cell types can be modified based on the presence or absence of these intercellular interactions. Oocyte maturation occurs in the interval between the first and the second meiotic block and is triggered by the preovulatory peak of LH that promotes the evolution of the dominant follicle to a preovulatory follicle. As previously stated, the CCs transmit gonadotropin-dependent signals to the oocyte, thereby exerting control over its development [149]. Proper maturation presupposes the acquisition of competence by the oocyte, which can support the various stages of development, such as fertilization and subsequent implantation of the embryo. In response to LH stimulation, an increase in intracellular Ca^2+^ has been documented to occur in the CCs [150] and then diffuse to the oocyte [151]. As for Ca^2+^, there is various evidence that confirms the function of Ca^2+^ in regulating oocyte maturation. In fact, one of the potential targets of Ca^2+^ in the oocyte appears to be adenylate cyclase (AC). Therefore, Ca^2+^ can be transferred from the CCs to the oocyte via gap junctions, where it can inhibit adenylate cyclase isoform III (AC3), leading to a decrease in the level of cAMP and thus a resumption of meiosis. Alternatively, Ca^2+^ can trigger CAMKII, which can then activate the meiotic maturation-promoting factor (MPF) or, alternatively, MAPK. MPF controls cell cycle progression [152], while MAPK is associated with microtubule organization, spindle formation, and chromosome separation in meiosis [153,154,155]. In contrast, the target of Ca^2+^ in CCs is not known. It is thought that the increase in Ca^2+^ due to LH release in CCs could activate the calcium-sensitive adenylate cyclase isoform I (AC1) [156], which in turn would turn on the cAMP pathway by expanding the action of FSH/LH. Alternatively, it could activate the MAPK pathway in CCs that is involved in estrogen and progesterone synthesis [157,158]. In addition, MAPK induces the expression of EGF-like factors [159,160] that, via their respective EGFR on CCs, act by positively influencing oocyte maturation (Figure 5). To demonstrate this, any removal of Ca^2+^ from the extracellular fluid or the buffering of its intracellular levels impairs oocyte maturation [161]. As a consequence of stimulation by LH, there is a progressive reduction in membrane conductance to potassium, leading to depolarization of the membrane potential of CCs [162]. Kv channels are the main determinants of membrane potential that are modulated by the action of gonadotropins [147,162,163,164]. Depolarization of the CCs then rapidly extends to the oocyte, being these two cell types coupled by gap junction [147]. Given the presence of P/Q-type Ca^2+^ channels on the membrane of the oocyte [151,165], the depolarization event has an effect on voltage-dependent channels, inducing an increase in intracellular Ca^2+^ that could contribute to meiotic recovery.

### 4.4. Clinical Significance of Electro-Metabolic Coupling of COC

CC expansion is crucial for good oocyte quality. Several studies show that gene expression at the level of CCs can provide reliable markers for assessing embryo quality, although the correlation needs to be demonstrated more consistently [166]. Electro-metabolic uncoupling could be one of the causes of female infertility as it compromises the mitochondrial functionality essential for the acquisition of oocyte competence [167,168]. CC functionality is also important in assisted reproduction techniques. For example, animal studies show that a heat shock occurs during cryopreservation processes, which can affect intracellular calcium homeostasis. This event leads to an electro-metabolic uncoupling of the COC as the cation acts by activating the oocytes during the fertilization process [169]. Consequently, this could also influence the mitochondrial biogenesis of COC, essential for oocyte maturation and downstream events such as fertilization and intracytoplasmic sperm injection (ICSI) [169,170,171].

## 5. Ovarian Aging

Ovarian aging is characterized by changes in the quantity and quality of the oocyte pool over time, leading to a decline in female fertility [172]. In women, the oocyte pool created during intrauterine life is gradually depleted [173], and this process depends on two components such as the initial size of the follicular pool and the process of follicular atresia. Studies in mice have shown that mitochondria play a key role in these two events; therefore, they are potentially related to the ovarian aging process. However, the depletion of the follicular pool that occurs in ovarian aging is caused by the apoptosis of oocytes and surrounding follicular cells [174]. Mitochondria play a fundamental role [175,176,177,178] in this step because they are involved in cell survival and apoptosis [179]. In addition, the determination of follicular pool size occurs during embryonic life along with mitochondrial biogenesis [180]. Ovarian aging appears to be related to quantitative and qualitative mitochondrial dysfunction. Quantitative dysfunction involves mtDNA copy number and mtDNA deletions, while qualitative dysfunction includes strand breaks, point mutations, and oxidative base damage. Mitochondrial DNA mutations and mitochondrial dysfunction in CCs could be related to oocyte maturity [181]. In older women with diminished ovarian reserve (DOR), the number of abnormal mitochondria in CCs increases compared to young women with normal ovarian reserve (NOR) [110]. This suggests that, with aging, also ATP production could decrease because of the altered mitochondrial activity and interfere with embryo development [107]. Based on this consideration, substances like resveratrol that affect mitochondrial biogenesis could have a positive impact on ovarian aging. The oocyte, being located within follicular cells, develops as an integral part of an ovarian microenvironment, consisting of both CCs and follicular fluid, and it can influence its quality and quantity [182]. Recently, it has been observed that the ovarian microenvironment is subject to changes that can be studied to obtain information on reproductive aging. For example, with age, in CCs, the copy number of mtDNA undergoes a decrease [183], and it is more likely to accommodate deletions [184]. Furthermore, transcriptome analysis of CCs in mice revealed [185] age-dependent changes, showing an over-expression of those genes involved in hypoxia stress response, angiogenesis, DNA damage/repair, and glycolysis in the elderly [185]. However, over-regulation of these genes may represent a compensatory response to follicular stress due to a potential suboptimal environment (e.g., hypoxia). In addition, the over-expression of proteins involved in fatty acid metabolism and downregulation of proteins involved in OXPHOS is also observed in CCs [186]. Also, follicular fluid tends to change with age, revealing altered levels of VEGF, an angiogenic protein that may play a role in reproductive aging [187,188,189,190,191,192]. The oocyte, being distant from the blood circulation, depends on oxygen diffusion through the surrounding GCs and follicular fluid. Follicular microvascularization appears to be regulated by angiogenic factors such as VEGF, which is produced by GCs with subsequent secretion and transport into the follicular fluid [193,194,195]. Several studies have highlighted that VEGF increases in the follicular fluid of older women, making it a biomarker of follicular hypoxia [187]. The metabolomic profile is also altered as a consequence of the aging process. In fact, there is an increase in glycolytic activity in CCs, probably to resist the follicular stress given by hypoxia and increased ROS. This altered metabolomic profile affects the expression of sirtuins, proteins that are involved in epigenetic modifications, regulation of mitochondrial function, and DNA repair and recombination [66,67]. In particular, one study showed that the levels of the SIRT3 and SIRT5 transcripts [69] and their activity decreased in the CCs and GCs of aged women with diminished ovarian reserve [69]. One of the primary mechanisms underlying the process of female reproductive aging may be the accumulation of ROS levels accompanied by a reduction in antioxidant capacity [81,196,197,198]. This has been observed not only in oocytes but also in cells of the CC, GC, and follicular fluid [199,200]. The formation of ROS with aging causes an alteration of the redox balance, resulting in the initiation of apoptosis, a highly regulated process that underlies follicular atresia [201,202]. During in vitro fertilization, the incidence of apoptosis in GCs [203] but also in CCs is positively correlated with age and is associated with decreased fertilization in women older than 40 years [204]. Therefore, this suggests that the vitality of CCs determines oocyte quality [204], although it is still not completely clear whether apoptosis may be a cause or a consequence of the decrease in oocyte quality with age. Cellular hypoxia is a phenomenon associated with aging. In fact, ovarian vascularization becomes deficient, and this contributes to a reduction in the expression of mitochondrial genes, which are important for ATP production, apoptosis, and Ca^2+^ homeostasis [205,206]. Therefore, the mitochondrial number is crucial for proper oocyte maturation, fertilization, and embryo development [205,206,207,208,209], and its reductions can be a major cause of infertility [210,211]. During hypoxia, the expression of the hypoxia-inducible factor HIF-1α turns out to be stable. This transcription factor promotes the induction or reduction in expression of genes involved in various cellular functions such as oxygen homeostasis, angiogenesis, cell survival, glucose metabolism, and apoptosis [212,213]. For example, HIF-1α regulates transcription of the gene encoding for VEGF factor by binding to hypoxia response elements [214,215]. The expression of HIF-1 is inhibited by some flavonoids [216,217].

## 6. Conclusions

**Figure 6:**
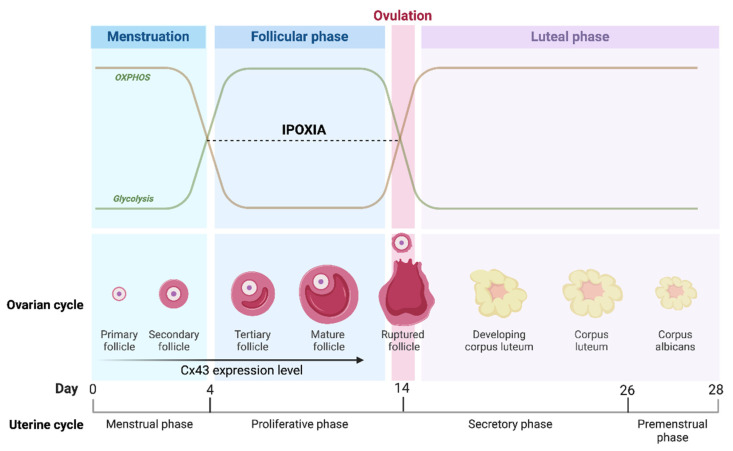
Metabolic switch in COC depending on the stage of folliculogenesis. The schematic diagram shows the metabolic switch of the COC in relation to follicle size depending on the stage of folliculogenesis. However, the metabolism of CCs is oxidative when the follicle is small and well irrigated, while it becomes glycolytic when the follicle grows and matures. Cx43 levels during the folliculogenesis indicate the formation of the maximum number of gap junctions when the follicle is mature. Created with BioRender.com (accessed on 25 January 2024).

Considering these preliminary observations, it would be interesting to treat CCs with resveratrol or other substances capable of modulating potassium currents to promote intracellular Ca^2+^ influx and consequently increase mitochondrial biogenesis and the resulting ATP levels. It can be hypothesized that resveratrol, affecting the electro-metabolic coupling between the cells of the oophorous cumulus and the oocyte, could have a significant positive effect on CCs, from which an improvement in ovarian physiology and oocyte development could be of benefit altogether. The modulation of electro-metabolic coupling of COC by resveratrol could explain the beneficial effect on fertility of women undergoing IVF [218,219,220,221]. It can also be hypothesized that the role of mitochondria present in the CCs may affect the maturation and competence of the oocyte, especially in the early stages of folliculogenesis, since the primordial follicle appears to be small and well irrorated. Therefore, the cumulus mitochondria, being in the presence of oxygen, produce a large amount of energy via OXPHOS. As the follicle grows and matures, the distance between the irrorated thecal layer and the COC increases, and oxygen diffusion becomes limited. Indeed, we understand from the literature that these two cell types reprogram their metabolism; in fact, the oocyte becomes more oxidative while the CC acquires a glycolytic phenotype. The COC meets oxygen again at the time of ovulation when after being released from the Graaf follicle, it enters the oviduct. Therefore, we advance the hypothesis of the existence of a dynamic window that promotes the shift of the metabolism from oxidative to glycolytic and vice versa, depending on the oxygen tension and the stage of folliculogenesis (Figure 6).
